# Embryonic Developmental Temperatures Modulate Thermal Acclimation of Performance Curves in Tadpoles of the Frog *Limnodynastes peronii*

**DOI:** 10.1371/journal.pone.0106492

**Published:** 2014-09-02

**Authors:** Frank Seebacher, Veronica S. Grigaltchik

**Affiliations:** School of Biological Sciences, University of Sydney, Sydney, New South Wales, Australia; Institute of Hydrobiology, Chinese Academy of Sciences, China

## Abstract

Performance curves of physiological rates are not fixed, and determining the extent to which thermal performance curves can change in response to environmental signals is essential to understand the effect of climate variability on populations. The aim of this study was to determine whether and how temperatures experienced during early embryonic development affect thermal performance curves of later life history stages in the frog *Limnodynastes peronii*. We tested the hypotheses that a) the embryonic environment affects mean trait values only; b) temperature at which performance of tadpoles is maximal shifts with egg incubation temperatures so that performance is maximised at the incubation temperatures, and c) incubation temperatures modulate the capacity for reversible acclimation in tadpoles. Growth rates were greater in warm (25°C) compared to cold (15°C) acclimated (6 weeks) tadpoles regardless of egg developmental temperatures (15°C or 25°C, representing seasonal means). The breadth of the performance curve of burst locomotor performance (measured at 10, 15, 20, 25, and 30°C, representing annual range) is greatest when egg developmental and acclimation temperatures coincide. The mode of the performance curves shifted with acclimation conditions and maximum performance was always at higher temperatures than acclimation conditions. Performance curves of glycolytic (lactate dehydrogenase activities) and mitochondrial (citrate synthase and cytochrome c oxidase) enzymes were modulated by interactions between egg incubation and acclimation temperatures. Lactate dehydrogenase activity paralleled patterns seen in burst locomotor performance, but oxygen consumption rates and mitochondrial enzyme activities did not mirror growth or locomotor performance. We show that embryonic developmental conditions can modulate performance curves of later life-history stages, thereby conferring flexibilty to respond to environmental conditions later in life.

## Introduction

Temperature influences physiological rate processes, and the temperature breadth across which animals can perform will determine their distribution and abundance across environments [1]. Performance typically increases gradually with increasing temperature until a maximum is reached, beyond which performance decreases as temperatures increase so that performance curves often have an inverted “U” shape [2]. Fitness may be compromised as temperatures experienced by animals diverge from those at which optimal performance occurs, thereby leading to population declines [3], [4]. However, performance curves of physiological rates are not fixed and may change in response to changing environmental conditions independently from genotypic changes [5], [6]. Determining the extent to which thermal performance curves can change within individuals in response to environmental signals is therefore essential to understanding the effect of climate variability on populations [7], [8].

Conditions experienced during embryonic development can influence physiological responses to the environment [9]–[12]. Such developmental plasticity can be advantageous if early developmental conditions predict the environmental conditions experienced at later life-history stages with the result that performance is maximised at the predominant environmental conditions experienced by offspring [13]. Developmental plasticity can be maladaptive if the environment experienced by offspring is different from embryonic developmental conditions [14]. Additionally, embryonic developmental conditions can also influence the capacity of offspring to respond to thermal variability in their environment by reversible thermal acclimation [15]–[17]. Hence, developmental conditions can influencing both mean trait values as well as their plasticity. Thermal plasticity induced by developmental or later acclimation conditions can manifest as a horizontal or vertical shift in the performance curve so that the temperature at which optimal performance occurs changes, or as increases or decreases in maximal performance, respectively. Additionally, plasticity can result in a change of the shape of the reaction norm, either increasing or decreasing the breadth [18], [19].

The aim of this study was to determine whether and how temperatures experienced by during early embryonic development in the frog (*Limnodynastes peronii*) affect thermal performance curves of later life history stages. Our study follows a three-factor design with egg incubation temperature, tadpole acclimation temperature, and acute test temperatures as factors. We tested the hypotheses [18] that a) the embryonic environment affects mean trait values only (incubation temperature main effect, no interactions); b) temperature at which performance of tadpoles is maximal shifts with egg incubation temperatures so that performance is maximised at the incubation temperatures (incubation×test temperature interaction); c) incubation temperatures modulate the capacity for reversible acclimation in tadpoles (incubation×acclimation temperature interaction). Where possible, that is where we were able to determine the performance maxima, we also analysed performance curves to determine whether early embryonic or later acclimation temperatures changed the breadth, mode, or maxima of the performance curves [20].

We chose the striped marsh frog (*Limnodynastes peronii*) as study organism, because tadpoles of *Lim. peronii* are known to acclimate swimming performance and metabolism [21], [22]. Also, the species occurs across a broad latitudinal range along the east coast of Australia [23], [24], and breeds for most of the year even at mid latitudes [25], [26]. Embryonic development of *Limnodynastes* in the egg is fairly rapid (3–5 days) but the tadpole stage is prolonged and variable between individuals (60–300 days) [27]. Tadpoles hatched late in summer are likely to overwinter and metamorphose in the next spring. During overwintering animals grow slowly [26] and there would be no demand to maximise physiological performance such as metabolic rates.

We were particularly interested in measuring performance curves of physiological responses across different levels of organisation. Hence, we measured the activities of enzymes that are important in mitochondrial (citrate synthase and cytochrome c oxidase) and glycolytic (lactate dehydrogenase) ATP-producing pathways [22], [28], [29]. Enzymes can become limiting if their maximal activities cannot supply sufficient ATP for locomotion and growth. We also measured oxygen consumption to determine ATP production underlying growth rates. Lastly, we measured swimming speed and growth rates, which are whole-animal performance measures that depend on metabolism and which are closely related to fitness [30], [31].

## Materials and Methods

### Ethic statement

Animals were collected under NSW National Parks and Wildlife Service Scientific License SL100518. All experiments were conducted under approval from the University of Sydney Animal Ethics Committee (approval L04/5-2011/2/5530).

### Study animals and acclimation

Freshly-laid egg masses (n = 6) of striped marsh frogs (*Limnodynastes peronii*) were collected from ponds (mean water temperatures at time of collection 19.5±0.5°C) in the Sydney, Australia, metropolitan area (33°78′ S; 151°26′ E). Ponds were checked in the evening and eggs were collected early morning to ensure that nests were laid that night. Eggs were transported to the University of Sydney and each egg mass was divided between two developmental treatments: a “warm” temperature treatment (water temperature: 25.0±0.5°C) and a “cold” temperature treatment (water temperature: 15.0±0.5°C). These temperatures were chosen because they represent mean conditions at the warm midpoint and cool beginning of the breeding season at this location, respectively [17]. We will refer to these treatments as the “incubation temperatures”.

**Figure 1:**
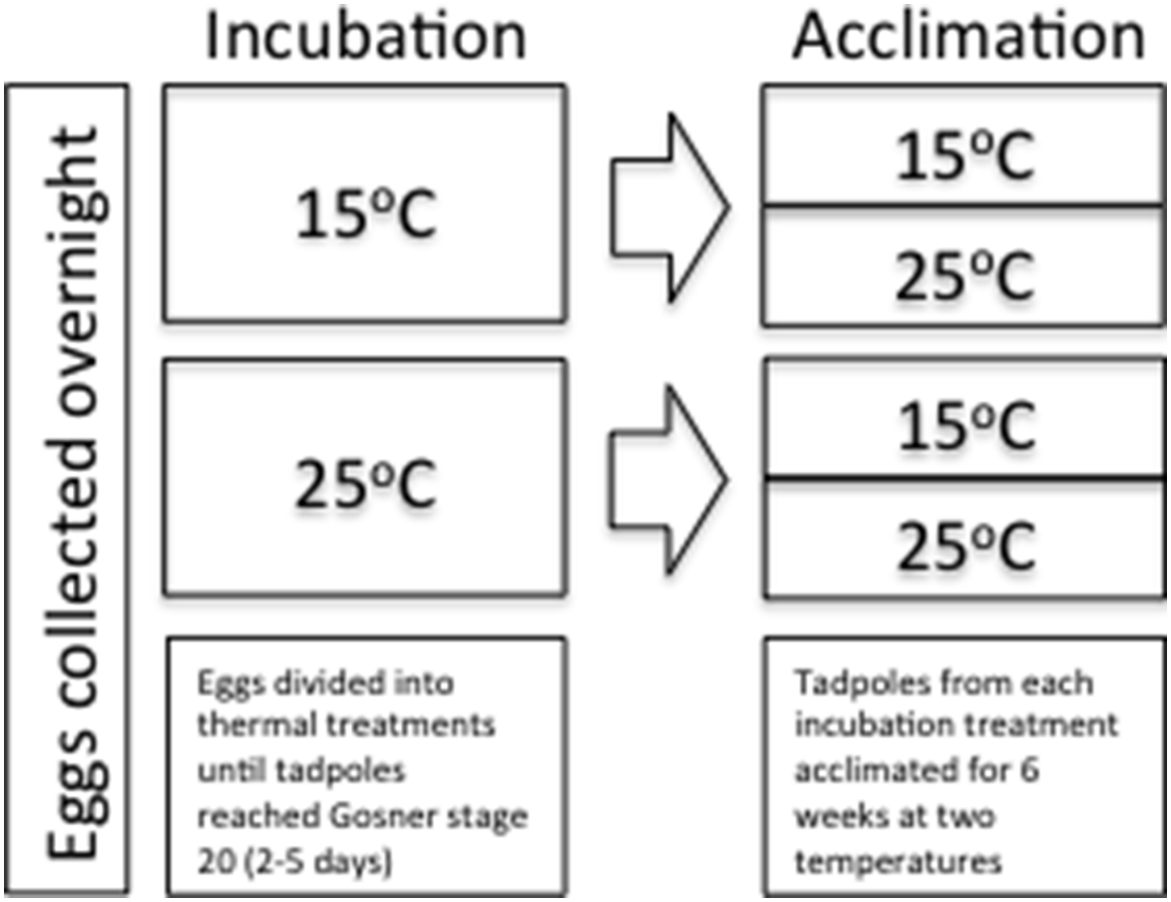
Flowchart summarising the experimental protocol. Eggs were collected from nests laid overnight and immediately divided into two (15°C and 25°C) incubation treatments until tadpoles reached Gosner stage 20 (after 2–5 days). Tadpoles from each incubation treatment were then acclimated to either 15°C or 25°C for six weeks.

When the tadpoles had hatched and reached Gosner stage 20 [32] (within 2–5 days), individuals were randomly selected from each developmental treatment and either acclimated to 15°C or 25°C, that is tadpoles from each nest were distributed across all experimental treatments to avoid clutch effects (Fig. 1). Hence, we had four treatment combinations: tadpoles from 15°C and 25°C incubation temperatures each acclimated to both 15°C and 25°C (Fig. 1). Acclimation temperatures were reached by increasing or decreasing water temperatures by 2.5°C per day, and animals were acclimated at their final treatment temperatures for 6 weeks. Within each acclimation treatment, we split tadpoles between six tanks (250×350×150 mm) with 60 tadpoles per tank. For each of the experimental measurements (see below), we used equal numbers of tadpoles from each tank within each treatment to minimise tank effects. Tadpoles were kept at a 12∶12-h light-dark cycle throughout the experiments and were fed frozen and thawed lettuce *ad libitum*. All trials were conducted during daylight hours and tadpoles were not fed for 24 hours before experimental measures were taken.

### Growth rates

Three tadpoles were taken from each tank every week (n = 18/treatment/week, except for the 25°C incubation×15°C acclimation treatment where there were only 15 tadpoles from weeks 3–6) throughout the acclimation period (6 weeks) and euthanised in a solution of buffered MS222 (0.25 g/L, pH 7.0; Sigma, Sydney, NSW, Australia). Total body length was measured with callipers to the nearest 0.1 mm, and the developmental (Gosner) stage was determined.

### Routine oxygen consumption

Tadpoles were gently dried, weighed (tadpoles did not have any yolk left at this stage) and placed into a plastic respirometer (43 ml). The respirometers were provided with an inflow and outflow port through which aged oxygenated water was supplied before measurements started, and which could be closed-off without disturbance to the animals. Inside each respirometer we placed an oxygen sensor spot (PSt3, PreSens, Regensburg, Germany) that was monitored with a fibre-optic cable (PreSens, Germany) mounted on the outside of the respirometer and connected to an oxygen meter (FIBOX3, PreSens, Germany).

After tadpoles were placed into the respirometers, the respirometers were left in a temperature controlled room set to the appropriate test temperature for at least one hour, which is sufficiently long for recovery from handling stress [33]. After this resting period, the respirometers were sealed and oxygen concentrations were determined every 15 minutes for a total of 60 minutes; we chose this approach rather than continuous readings to increase the rate at which we could measure tadpoles to ensure that all tadpoles from different treatments could be processed rapidly to minimise potential confounding effects of time. We used the slope in the decline in oxygen concentrations over the last 45 minutes of measurements to determine rates of oxygen consumption in µmol min^−1^g^−1^ wet mass [34]; oxygen consumption rates stabilised over that time period.

We used independent tadpoles (n = 18/treatment/test temperature, i.e. 3 from each tank) to measure rates of oxygen consumption at each of five test temperatures (10, 15, 20, 25 and 30±0.1°C; i.e. n = 90 tadpoles total per treatment), and tadpoles within each treatment were tested in random order. The test temperatures were chosen because they represent the thermal extremes experienced by tadpoles at this location. We also measured oxygen consumption in respirometers not containing tadpoles as controls, and we subtracted oxygen consumption by the controls from the experimental values. In the event, oxygen consumption by controls was kept to a minimum by routinely cleaning equipment and regular water changes. It was not possible to keep tadpoles stationary during trials so that the measurements represent routine rates of oxygen consumption that represent a mixture of oxygen consumed during low levels of voluntary activity, and maintenance and growth.

### Burst swimming performance

To measure burst swimming speed, individuals were placed into a plastic tray (40 cm×25 cm×5 cm) filled with water to a depth of 2 cm and were allowed to equilibrate for 15 minutes; trays were kept in constant temperature rooms set at the desired experimental temperature, and water temperature was allowed to equilibrate to room temperature before measurements. The tray dimensions were large relative to the distance moved during an escape response so that data were not confounded by edge effects. Individuals were within the range of developmental stages (Gosner 25–30) in which the relationship between total body length and burst swimming performance remains constant [21]. Burst swimming responses were initiated by lightly tapping the tail to elicit an escape response [35]. The escape responses were filmed from above with a camera (Quickcam Pro, Logitech, China) filming at 30 frames per second. Three responses were filmed for each individual, and videos were analysed in video analysis software (Tracker, Open Source Physics, USA) to determine the maximum speed; we used the maximum speed attained during all three escape response as burst speed in the analysis. As for oxygen consumption, we used independent tadpoles (n = 18/treatment/test temperature; total n = 90 tadpoles/treatment) to measure burst speed at each of five test temperatures (10, 15, 20, 25 and 30±0.1°C) in random order. Swimming speed is presented and analysed as body lengths s^−1^ (BL s^−1^).

### Enzyme assays

After euthanasia in a solution of buffered MS222 (0.25 g/L, pH 7.0; Sigma, Sydney, NSW, Australia), tadpoles (n = 9/treatment; Gosner stages 30–39) were weighed, measured, and their developmental stage was determined before we dissected axial tail muscle. Tail muscle was frozen at −80°C for later analysis. We measured actitivites (V_max_) of lactate dehydrogenase (LDH), an indicator of glycolytic ATP production, and the two mitochondrial enzymes citrate synthase (CS) and cytochrome c oxidase (COX). Samples were homogenised in 1∶20 (CS and COX) or 1∶100 (LDH) w/v extraction buffer (50 mM imidazole/HCl, 2 mM MgCl_2_, 5 mM ethylene diamine tetra-acetic acid (EDTA), 0.1% Triton X-100, and 1 mM glutathione, pH = 7.5). Enzyme assays were conducted at 10, 15, 20, 25, and 30°C according to published protocols [29].

### Statistical analysis

Growth rates, metabolic rates, and enzyme activities increased approximately linearly over time or test temperatures at which they were measured. Hence, we used a permutational analysis of variance (PERMANOVA; Primer 6, ePrimer, Plymouth, UK) to analyse differences between treatments. Permutational analyses use the data per se rather than an assumed underlying distribution for statistical inference. This approach is superior for laboratory-based experiments with relatively small sample sizes relative to the total population [36]. We analysed growth with incubation temperature (15°C and 25°C), acclimation temperature (15°C and 25°C), and week (1–6) as fixed factors. For all other analyses we used incubation temperature (15°C and 25°C), acclimation temperature (15°C and 25°C), and test temperature (10–30°C) as fixed factors.

Additionally, we analysed the performance curve of swimming speed explicitly to determine changes in performance breadth, maxima, and temperatures at which maxima occurred. We could conduct this analysis on swimming data only because it was the only response that was non-linear across the range of test temperatures (see below). Thermal reaction norms can be described by Gaussian (inverted “U”-shaped) distributions [20]. However, swimming performance was the only response variable that reached a maximum within the range of test-temperatures we used, and therefore the only one that permitted us to fit Gaussian functions. Metabolic rates and enzyme activities increased linearly over the range of test temperatures (see below).

Gaussian functions are in the form: Y = a*exp(−0.5((X−b)^2^/c^2^), where a = amplitude (maximum), b = mode that aligns the curve on the temperature (x) axis, and c = breadth of the curve. To compare these parameters between treatments, we performed a bootstrap analysis on our data to determine mean parameter values and 95% confidence intervals [37]. Bootstrap values were determined by firstly randomising swimming speed data within each test temperature, within incubation and acclimation temperature treatments. We then determined the mean of a subsample of swimming speed data (n = 6) at each test temperature to obtain a reaction norm to which we fitted a Gaussian curve in Prism 5.0 software (GraphPad Software, USA). This process was repeated 500 times for each of the four incubation and acclimation temperature treatment combinations, and we calculated the mean and 95% confidence intervals for the amplitude, mode, and breadth of the Gaussian curves from these 500 bootstrap values. We used the confidence intervals to infer differences in parameters between treatments [37].

## Results

### Growth

**Figure 2:**
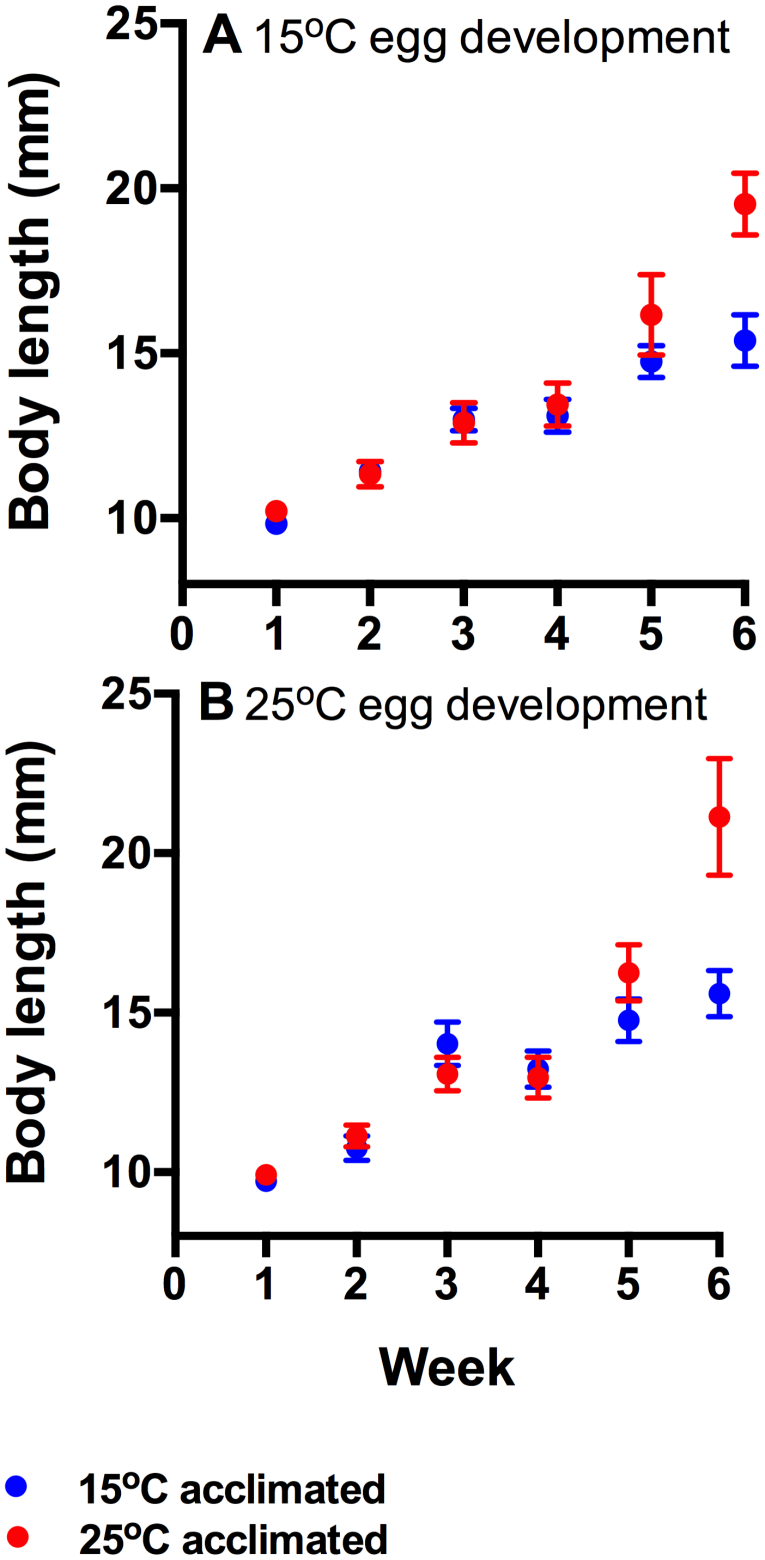
Growth (mean ± s.e.) in total length of tadpoles hatched from eggs incubated at 15°C (A) and at 25°C (B) that have subsequently been acclimated to 15°C (blue circles) or 25°C (red circles). Warm-acclimation tadpoles had significantly higher growth rates at weeks 5 and 6 (acclimation×time interaction), but incubation temperatures did not have a significant effect. N = 15–18 tadpoles per treatment group per week.

There was an interaction between acclimation temperature and time (F_5,419_ = 5.33, p<0.001; Fig. 2), and at the final week of measurement warm acclimated tadpoles were significantly longer than cold acclimated tadpoles regardless of incubation temperature. Incubation temperature did not have a main effect (F_1,419_ = 0.25, p = 0.673) and there were no interactions between incubation temperature and time (F_5,419_ = 0.51, p = 0.812) or acclimation temperature (F_1,419_ = 0.042, p = 0.933). At the end of the 6 week acclimation period, tadpoles from the 15°C incubation temperature on average reached Gosner stage 25.3±0.1 (s.e.) at 15°C acclimation temperature and 25.8±0.2 (s.e.) at 25°C acclimation temperature; tadpoles from the 25°C incubation temperature reached Gosner stage 25.4±0.1 (s.e.) after 6 weeks of acclimation to 15°C, and 27.6±1.4 (s.e.) after acclimation to 25°C.

### Burst swimming speed

Incubation temperature had a significant main effect on swimming speed (F_1,340_ = 7.29, p<0.01), and tadpoles that developed from eggs incubated at 15°C had higher swimming performance (12.39 BL s^−1^ ±0.34 s.e.) than tadpoles from eggs incubated at 25°C (11.47 BL s^−1^ ±0.35 s.e.). Additionally, there was a significnat interaction between acclimation and test temperatures (F_4,340_ = 12.24, p<0.001). None of the other interactions were significant (all F<2.0, p>0.1).

**Figure 3:**
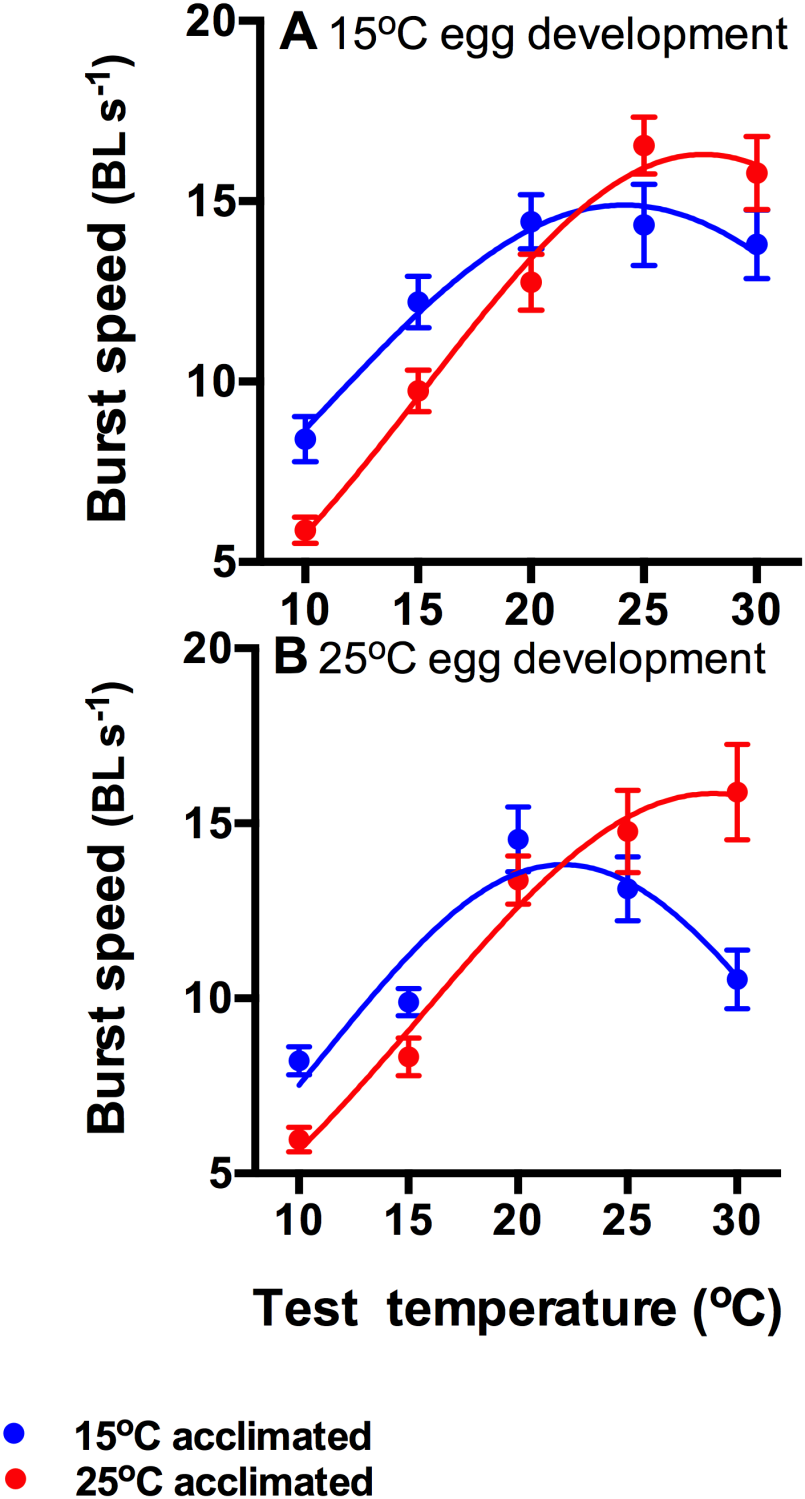
Burst swimming speed (mean ± s.e.) of tadpoles hatched from eggs developed at 15°C (A) and at 25°C (B) that have subsequently been acclimated to 15°C (blue circles) or 25°C (red circles). The lines show Gaussian reaction norms fitted to the data from cold (blue lines) and warm (red lines) acclimated animals. The amplitudes, breadths and modes of the reaction norms were determined by an interaction between incubation and acclimation temperatures (Table 1). N = 18 tadpoles per treatment and test temperature.

Thermal reaction norms (Table 1; Fig. 3) of tadpoles acclimated to 25°C had the greatest amplitude regardless of incubation temperature, and maximum burst swimming performance was lowest in tadpoles hatched from warm eggs and acclimated to 15°C (Fig. 3B). The mode of the reaction norms varied considerably (by 8.3°C) between treatments. Tadpoles incubated at 25°C but acclimated to 15°C had the lowest mode (Fig. 3B), followed by tadpoles incubated and acclimated to 15°C (Fig. 3A). Tadpoles incubated and acclimated to 25°C had the highest mode (Fig. 3B; Table 1). Interestingly, performance breadth was greatest when incubation and acclimation temperatures coincided, and lowest in tadpoles that developed at 25°C but were acclimated to 15°C (Table 1).

**Table 1:** Bootstrap means and 95% confidence intervals (+/− CI) of amplitude (AMP), Mode, and Breadth of the Gaussian equations describing thermal reaction norms of swimming performance of tadpoles from the different treatments (TRT).

TRT	AMP	−CI	+CI	Mode	−CI	+CI	Breadth	−CI	+CI
15×15	15.11	15.05	15.16	24.77	24.59	24.95	14.10	13.88	14.31
15×25	16.69	16.61	16.77	28.40	28.20	28.59	12.67	12.54	12.80
25×15	14.01	13.95	14.06	22.22	22.15	22.29	11.04	10.93	11.15
25×25	16.82	16.64	16.99	30.50	30.11	30.88	13.81	13.58	14.03

In the Treatment column, the first number indicates developmental temperatures, the second acclimation temperatures.

### Oxygen consumption

**Figure 4:**
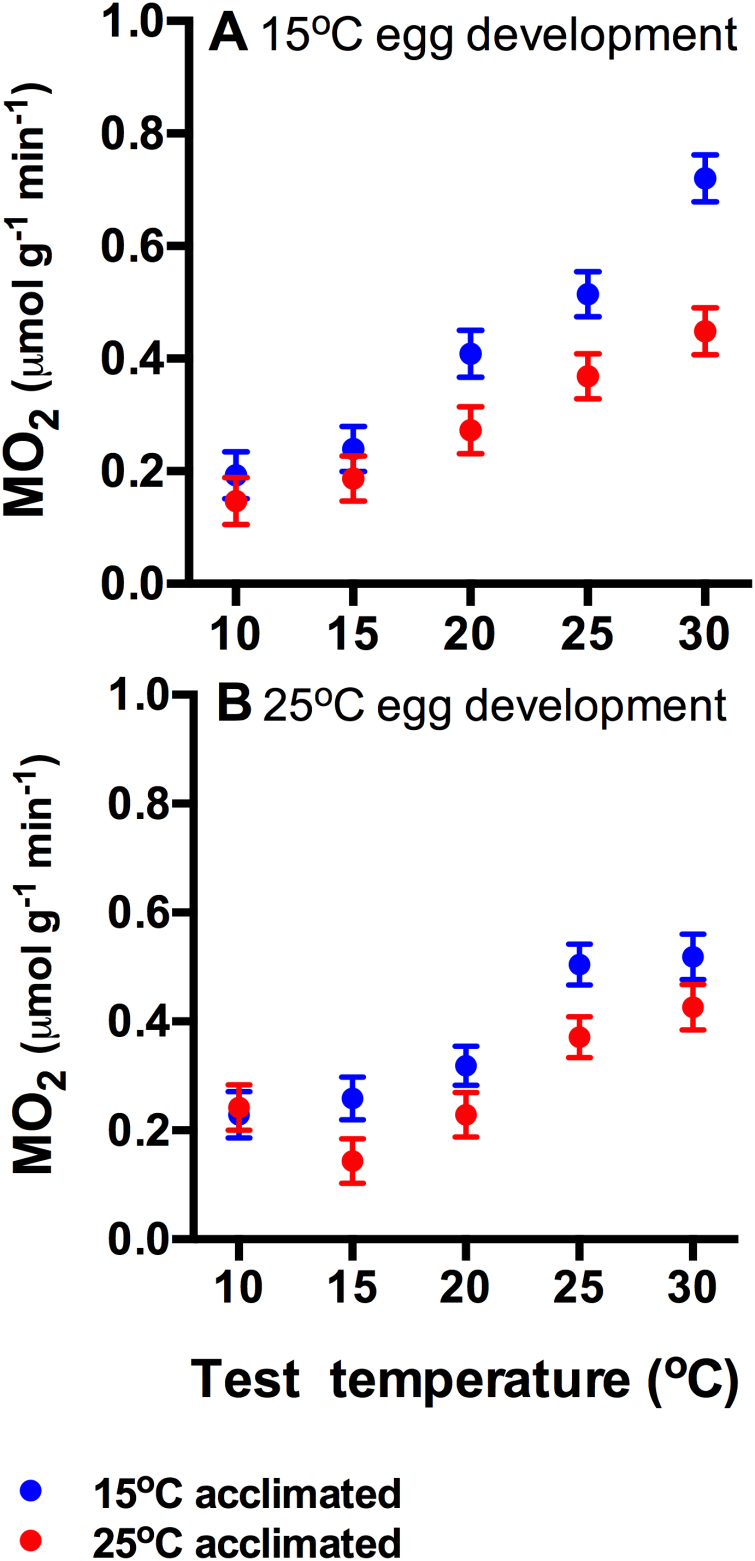
Routine metabolic rates (mean ± s.e.) of tadpoles hatched from eggs developed at 15°C (A) and at 25°C (B) that have subsequently been acclimated to 15°C (blue circles) or 25°C (red circles). Cold-acclimated tadpoles had significantly higher metabolic rates particularly at higher test temperatures (acclimation×test temperature interaction), but incubation temperatures did not have a significant effect. N = 18 tadpoles per treatment and test temperature.

Cold acclimated tadpoles had significantly higher routine oxygen consumption rates, particulalry at higher test temperatures (interaction between acclimation temperature and test temperature: F_4,385_ = 2.45, p<0.05; Fig. 4). Incubation temperature did not affect routine oxygen consumption rates (main effect: F_1,385_ = 0.86, p = 0.40; interactions between incubation temperature and acclimation temperature: F_1,385_ = 1.75, p = 0.17; and test temperatures: F_4,385_ = 1.79, p = 0.058).

### Enzyme activities

**Figure 5:**
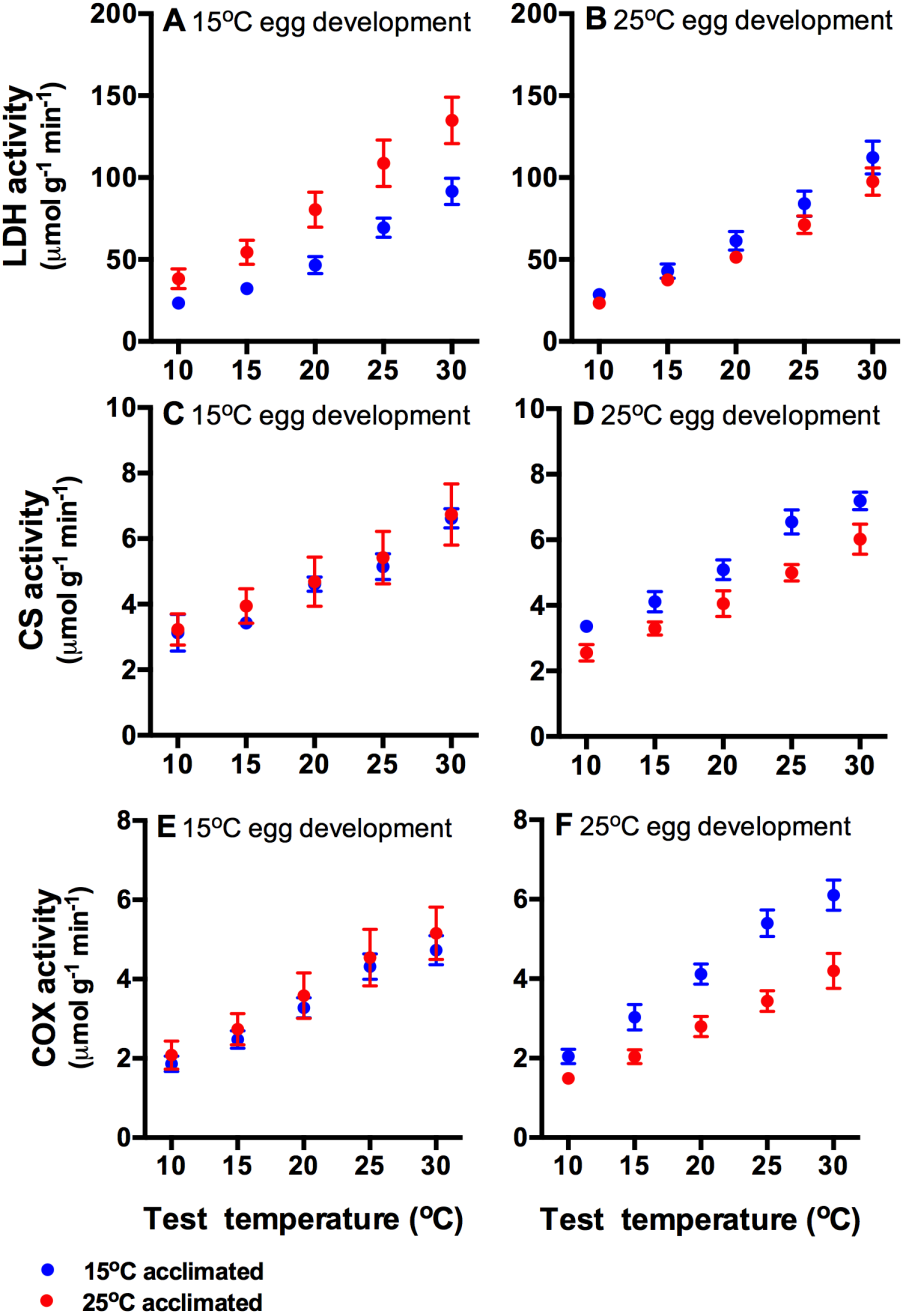
Activities of enzymes (mean ± s.e.) important for glycolytic (lactate dehydrogenase, LDH [A, B], and mitochondrial (citrate synthase, CS [C, D]; cytochrome c oxidase, COX [E, F]) energy (ATP) production. Data from eggs incubated at 15°C are shown in the left panels, and those from eggs incubated at 25°C are shown in the right panels. After hatching, tadpoles were acclimated to either 15°C (blues circles) or 25°C (red circles). Enzyme activities were determined by interactions between incubation and acclimation temperatures (see text for details). N = 9 tadpoles per treatment.

Lactate dehydrogenase activity was determined by a three-way interaction between incubation temperature, acclimation temperature, and test temperature (F_4,179_ = 2.51, p<0.05; Fig. 5A,B). Warm acclimated tadpoles from the cold incubation temperature in particular had the greatest LDH activity.

Citrate synthase activity was similar in all tadpoles from 15°C incubated eggs, but cold acclimated tadpoles had higher activity than warm acclimated animals from eggs incubated at 25°C (interaction between incubation temperature and acclimation temperature: F_1,179_ = 9.05, p<0.001; Fig. 5C, D). There was no interaction between test temperautre and incubation or acclimation temperatures (both F_4,179_ = 0.60, p>0.75).

Similar to citrate synthase, cytochrome c oxidase activity of 15°C acclimated tadpoles that were incubated at 25°C had greater activity than 25°C acclimated animals, but there were no differences in tadpoles incubated at 15°C (interaction between incubation temperature and acclimation temperature: F_1,179_ = 17.05, p<0.001; Fig. 5E, F). There was no interaction between test temperature and incubation or acclimation temperature (both F_4,179_<1.00, p>0.45).

## Discussion

Incubation temperature changed the mean trait values of swimming performance (hypothesis a; incubation main effect), but not those of any other traits. Additionally, acclimation of tadpoles changed the mode and breadth so that curves for swimming performance were determined by a combination of thermal conditions experienced by embryos and tadpoles. However, we found no evidence in any trait that incubation temperatures shift performance curves so that temperatures at which performance maxima of tadpoles occur coincide with egg incubation temperatures (hypothesis b; incubation×test temperature interaction). Incubation temperatures did modulate acclimation of performance curves of enzyme activities (hypothesis c; incubation×acclimation interactions). Lastly, growth was the only response that was independent from incubation conditions and tadpoles from warm acclimation treatments grew at a faster rate.

Locomotor performance was the only trait that showed a non-linear response to acute temperature increases across our range of test temperatures. Hence, unlike locomotor performance, growth, oxygen consumption and enzyme activities were maximal at temperatures above 30°C. Mean environmental conditions at the study site are below 30°C [17] indicating that high temperatures do not constrain these traits under current climate conditions. However, it is important to note that maximal rates do not necessarily reflect optimal rates. Increasing metabolic rates, for example, also translate into greater demand for food which may pose an ecological constraint. The fact that maximal swimming performance occurred within the test temperature range meant that we could analyse the shape of the performance curve in more detail. Tadpoles that were incubated and acclimated to 15°C had the greatest performance breadth but a relatively low maximum. In their natural environment, tadpoles experiencing 15°C as eggs either would hatch early in the breeding season and then experience gradual warming during their tadpole stages, or very late in the season and remain at the low temperature. Interestingly, responses of tadpoles that were incubated at 15°C and then acclimated to 25°C showed the opposite direction, that is relatively narrow performance breadth but a high maximum. If tadpoles experienced environments that spanned this whole temperature range, that is incubation at 15°C with later tadpole stages experiencing 25°C, animals would transition from thermal generalists during early Gosner stages to thermal specialist at later stages. Such environmental conditions are likely to occur commonly in ponds and stream. The plasticity of reaction norms may be advantageous to support the relatively inactive early stages in the variable and warming environment, while optimising burst speed of later stages to the prevailing warm conditions. Tadpoles that were incubated and grew to metamorphosis at the same warm conditions performed best both with respect to maximum and breadth of burst swimming. However, tadpoles developed from eggs incubated at warm temperatures, but which subsequently experienced cool conditions, as would be the case in late summer when temperatures were still high, had the lowest maximum and narrowest performance breadth of all four scenarios. These animals grow slowly and usually overwinter [26] to metamorphose during the following spring or summer. Interestingly, the temperature at which maximum burst speed occurred was considerably higher than the long-term acclimation temperature experienced by tadpoles. This means that animals do not perform at their maximum in their average thermal environment. An observation worth emphasising is that the mode of the reaction norms shifted with incubation and acclimation conditions so that there is no species-specific optimum temperature.

Egg incubation temperature did not affect growth rates of tadpoles, which is a similar pattern as in the woodfrog (*Rana sylvatica*) [15]. In contrast to *Rana*, however, in *Lim. peronii* tadpoles the fastest growth rates occurred in animals that experienced high temperatures towards the later developmental stages. Under natural conditions, this would be tadpoles that hatched from eggs laid early in the season but subsequently experience warm temperatures, or from those laid during summer. Tadpoles hatching from eggs laid late in summer when temperatures are still high but then cool or those laid late in the season at low temperatures will grow slowly and overwinter. These latter animals also have the slowest locomotor performance. Hence, the interaction between incubation and acclimation temperatures produced locomotor phenotypes that are responsive to variation in environmental conditions and are matched to growth patterns.

The metabolic responses of *Lim. peroni* tadpoles are not straightforward to reconcile with growth patterns. The high oxygen consumption rates of cold-acclimated tadpoles (regardless of incubation temperatures) indicates that oxygen consumption, and hence mitochondrial flux, is not driven predominantly by growth as would have been expected for larval animals. Cold-acclimated tadpoles were therefore either more active than warm acclimated animals, or more oxygen was used by cold-acclimated tadpoles for a given level of activity. An alternative is that activity at low temperature requires more ATP per unit muscle power output than at high temperature. Isolated gastrocnemius muscle from the African clawed frog *Xenopus laevis* used more oxygen at low (15°C) than at high (25°C) acute temperatures per Joule of work performed regardless of acclimation conditions. The reason for this difference is at least partly that the stiffness of the muscle is greater at low temperature and therefore requires greater cross-bridge activity to achieve force production [38]. If this were a general pattern among amphibians, it could explain the patterns we observed in *Lim. peronii* tadpoles. Additionally, cold acclimation can lead to increased proportions of oxidative fibres in fish [28], [39], and a change in muscle composition can explain increases in metabolic rate in response to cold acclimation [39].

Unlike growth and routine metabolic rates, incubation temperatures did affect the capacities for glycolytic and mitochondrial ATP production, and tadpoles from cross-over temperatures (cold incubation and warm acclimation or vice versa) had the greatest activities. The high lactate dehydrogenase activities of tadpoles developed from cool eggs but acclimated to warm temperatures matches their high burst swimming performance. Lactate dehydrogenase provides ATP quickly for short bursts of activity and it is commonly associated with burst performance [40]. Hence, in our tadpoles it may be a rate-limiting regulatory mechanisms, which is similar to patterns seen in the mosquitofish *Gambusia holbrooki* [17].

The high activities of the mitochondrial enzymes at low acclimation temperature are consistent with our possible explanations for increased metabolic rates above, that is increased ATP demand for a given muscle power output, and increased proportions of oxidative fibres. However, the pattern of high mitochondrial enzyme activities at cold acclimation temperatures was apparent only in tadpoles from eggs developed at 25°C. Mitochondrial enzyme activities do not predict swimming performance and growth rates. It would not be expected that burst swimming performance is constrained by mitochondrial activities [40]. Growth rates on the other hand rely on continuous, oxidative ATP production. However, the V_max_ of enzymes is not necessarily rate-limiting. Flux through metabolic pathways can be controlled by changes in substrate concentrations and allosteric regulators at numerous points in a pathway rather than by single rate limiting steps [41]. Incubation and acclimation thermal conditions can change the way in which pathways are regulated [17] and this could be an interesting area to explore further in tadpoles.

Developmental modification of phenotypes is increasingly recognised as a crucial mechanisms that underlies animal function [42], [43]. Relatively little is known about the function and mechanisms of developmental influences on phenotypes in animals other than medical models [44]. However, the mechanisms that establish developmental modifications appear to be conserved evolutionarily and it is likely that the processes in ectothermic animals are similar to that of mammals [43]. Pre-zygotic and early developmental conditions can influence phenotypes of later life-history stages by modifications of DNA molecules and histones [45]. Methylation at the five-carbon of cytosine bases on DNA molecules is a reversible process that can directly influence gene activity. Patterns of DNA methylation are established during development, and remain relatively stable through adult life. In contrast, the modification of histones is more reversible, and causes transcription of genes to be switched on and off [43], [45]. These mechanisms of DNA and histone modification are crucial for physiological function [44], [46], and discovering their role in mediating responses of ectotherms to environmental variability will be a significant advance in the field. A particularly interesting question would be how different environmental factors such as temperature and photoperiod [47]–[49] interact to determine developmental and reversible acclimation of physiological functions.
